# Methyl 3′,4′,5′-trimeth­oxy­biphenyl-4-carboxyl­ate

**DOI:** 10.1107/S1600536813004133

**Published:** 2013-02-16

**Authors:** Manu Lahtinen, Kalle Nättinen, Sami Nummelin

**Affiliations:** a University of Jyväskylä, Department of Chemistry, PO Box 35, FI-40014 JY, Finland; b VTT Technical Research Centre of Finland, Tampere, FIN-33101, Finland; c Molecular Materials, Department of Applied Physics, School of Science, Aalto University, PO Box 15100, FI-00076 Aalto, Finland

## Abstract

In the title compound, C_17_H_18_O_5_, the dihedral angle between the benzene rings is 31.23 (16)°. In the crystal, the mol­ecules are packed in an anti­parallel fashion in layers along the *a* axis. In each layer, very weak C—H⋯O hydrogen bonds occur between the meth­oxy and methyl ester groups. Weak C—H⋯π inter­actions between the 4′- and 5′-meth­oxy groups and neighbouring benzene rings [meth­oxy-C–ring centroid distances = 4.075 and 3.486 Å, respectively] connect the layers.

## Related literature

For a related structure, see: Li *et al.* (2012 ▶). For the nature of hydrogen bonding, see Steiner (2002 ▶); For related biphenyl structures, see: Leowanawat *et al.* (2011 ▶); Wilson *et al.* (2008 ▶); Percec *et al.* (2004 ▶); Suzuki (1999 ▶). For details of the synthesis and amphiphilic supra­molecular biphenyl dendrimers, see: Percec *et al.* (2006 ▶, 2007 ▶). For general background to self-assembling dendrons and dendrimers, see: Rosen *et al.* (2009 ▶); For the use of aromatic and aliphatic ester derivatives in the synthesis of dendrimers and dendrons, see Nummelin *et al.* (2000 ▶); Twibanire & Grindley (2012 ▶).
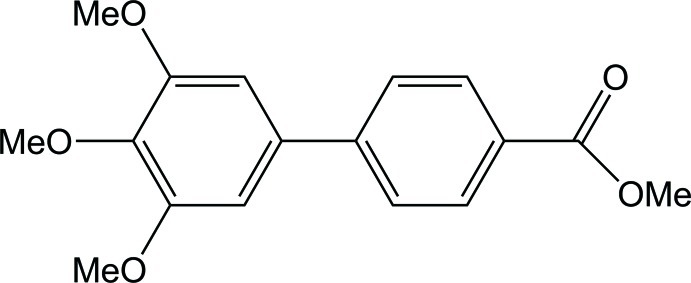


## Experimental

### 

#### Crystal data

C_17_H_18_O_5_
*M* *_r_* = 302.31
Triclinic, 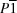

*a* = 7.9103 (5) Å
*b* = 8.6054 (7) Å
*c* = 11.8779 (7) Å
α = 92.834 (6)°
β = 92.448 (5)°
γ = 115.822 (7)°
*V* = 725.07 (9) Å^3^
*Z* = 2
Cu *K*α radiation
μ = 0.84 mm^−1^
*T* = 123 K
0.49 × 0.23 × 0.10 mm

#### Data collection

Agilent SuperNova (Dual source with Cu, Atlas) diffractometer
Absorption correction: analytical (*CrysAlis PRO*; Agilent, 2010) ▶ *T*_min_ = 0.827, *T*_max_ = 0.951
4547 measured reflections
2731 independent reflections
2524 reflections with *I* > 2σ(*I*)
*R*_int_ = 0.016

#### Refinement

*R*[*F* ^2^ > 2σ(*F* ^2^)] = 0.034
*wR*(*F* ^2^) = 0.096
*S* = 1.05
2731 reflections
204 parameters
H-atom parameters constrained
Δρ_max_ = 0.26 e Å^−3^
Δρ_min_ = −0.25 e Å^−3^

### 

Data collection: *CrysAlis PRO* (Agilent, 2010 ▶); cell refinement: *CrysAlis PRO*; data reduction: *CrysAlis PRO*; program(s) used to solve structure: *SUPERFLIP* (Palatinus & Chapuis, 2007 ▶); program(s) used to refine structure: *SHELXL97* (Sheldrick, 2008 ▶); molecular graphics: *OLEX2* (Dolomanov *et al.*, 2009 ▶) and *Mercury* (Macrae *et al.*, 2006 ▶); software used to prepare material for publication: *OLEX2*.

## Supplementary Material

Crystal structure: contains datablock(s) I, global. DOI: 10.1107/S1600536813004133/fj2614sup1.cif

Structure factors: contains datablock(s) I. DOI: 10.1107/S1600536813004133/fj2614Isup2.hkl

Supplementary material file. DOI: 10.1107/S1600536813004133/fj2614Isup3.cml

Additional supplementary materials:  crystallographic information; 3D view; checkCIF report

## Figures and Tables

**Table 1:** Hydrogen-bond geometry (Å, °)

*D*—H⋯*A*	*D*—H	H⋯*A*	*D*⋯*A*	*D*—H⋯*A*
C15—H15*A*⋯O2^i^	0.98	2.58	3.4933 (15)	156
C18—H18*C*⋯O14^ii^	0.98	2.50	3.4453 (16)	161

Symmetry codes: (i) 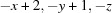; (ii) 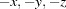.

**Fig. 1.**
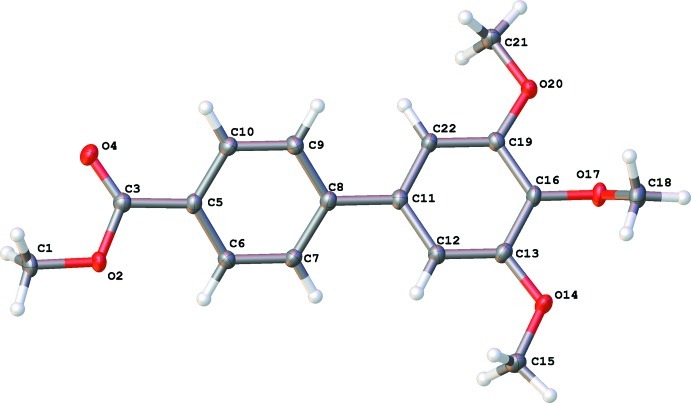
The asymmetric unit of (I) showing labeling scheme and displacement ellipsoids with 50% probability.

**Fig. 2.**
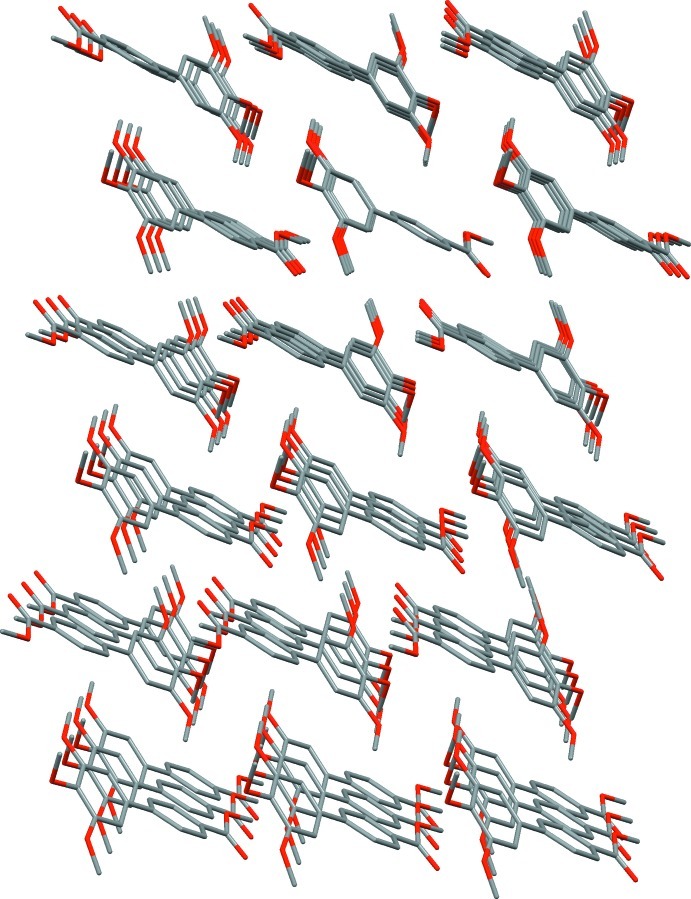
Molecular packing along a-axis. Hydrogen atoms are omitted for clarity.

**Fig. 3.**
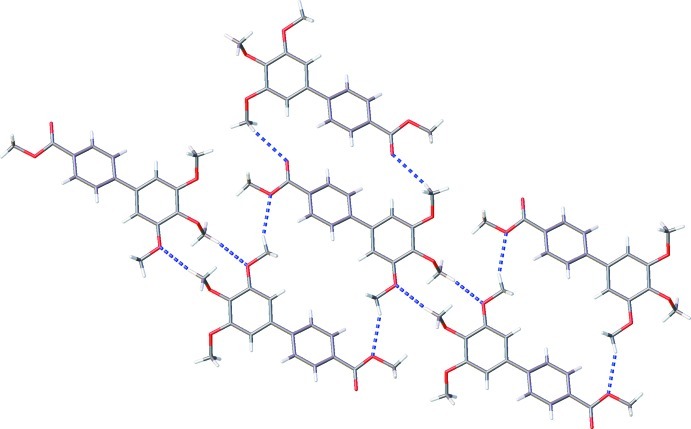
View of C–H···O contact network on a single molecule layer.

**Fig. 4.**
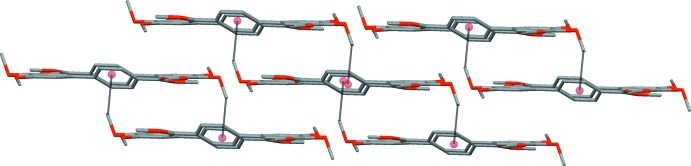
View along (1 - 2 2) plane showing weak C–H···π interactions between the layers of molecules.

## supplementary crystallographic information

### Comment

Aromatic and aliphatic ester derivatives are extensively used for the synthesis of various types of dendrimers and dendrons (Nummelin *et al.* 2000, Twibanire *et al.* 2012). In addition to arylmethyl ether-ester compounds, corresponding biphenyls are employed as building blocks for the construction of amphiphilic dendrons (Percec *et al.* 2006, 2007). These dendrons self-assemble into hollow and non-hollow supramolecular dendrimers that further self-organize into periodic assemblies (Rosen *et al.* 2009). The key step in the multi-step reaction sequence is a metal catalyzed aryl-aryl cross-coupling (Percec *et al.* 2004, 2006; Suzuki 1999). As a contribution to a structural study of biphenyl ether derivatives we report here the title compound methyl-(3',4',5'-trimethoxybiphenyl)-4-carboxylate (I).

Compound (I) crystallizes in triclinic space group *P*-1 (No. 2) without any solvent molecule in an asymmetric unit (Fig. 1). The intramolecular dihedral angle between the phenyl moieties is 31.23 (16)° [C7–C8–C11–C12]. The molecules are packed in antiparaller rows along *a*-axis (Fig. 2). On each layer of molecules, very weak C–H···O hydrogen bonds (Steiner 2002) are found between the methoxy and methyl ester groups d(H···A) varying from 2.5 to 2.6 Å (Fig. 3). The 4'-methoxy groups are pointing out from the otherwise planar molecules with bond and torsion angles of 121.07 (10)° and -72.99 (13)° [C13–C16–O17; C13–C16–O17–C18], respectively. In consequence of the projecting methoxy group and the 5'-methoxy group, the molecule layers are interconnected (Fig. 4.) *via* C–H···π interactions occurring between methoxy group H atoms and close by phenyl rings with distances of 4.075 and 3.846 Å [from methoxy(C) to phenyl ring centroid].

### Experimental

3',4',5'-trimethoxy-biphenyl-4-carboxylic acid (21.0 g, 72.8 mmol) was dissolved in MeOH (300 ml) and conc. sulfuric acid (3 ml). The solution was stirred under reflux for 14 h and then allowed to cool down to ambient temperature. The precipitate was collected by filtration, washed with water and dried in *vacuo* affording the title ester (20.4 g, 93%) as a white crystalline solid. Crystals suitable for a single-crystal structure determination were obtained from a slow evaporation of ethanol solution.

### Refinement

Hydrogen atoms were calculated to their positions as riding atoms (C host) using isotropic displacement parameters that were fixed to be 1.2 or 1.5 times larger than those of the attached non-hydrogen atom.

### Crystal data

**Table:**

C_17_H_18_O_5_	*Z* = 2
*M**_r_* = 302.31	*F*(000) = 320
Triclinic, *P*1	*D*_x_ = 1.385 Mg m^−^^3^
*a* = 7.9103 (5) Å	Cu *K*α radiation, λ = 1.5418 Å
*b* = 8.6054 (7) Å	Cell parameters from 3244 reflections
*c* = 11.8779 (7) Å	θ = 3.7–76.2°
α = 92.834 (6)°	µ = 0.84 mm^−^^1^
β = 92.448 (5)°	*T* = 123 K
γ = 115.822 (7)°	Plate, colourless
*V* = 725.07 (9) Å^3^	0.49 × 0.23 × 0.10 mm

### Data collection

**Table:**

Agilent SuperNova (Dual source with Cu, Atlas) diffractometer	2731 independent reflections
Radiation source: SuperNova (Cu) X-ray Source	2524 reflections with *I* > 2σ(*I*)
Mirror monochromator	*R*_int_ = 0.016
Detector resolution: 10.3953 pixels mm^-1^	θ_max_ = 70.0°, θ_min_ = 3.7°
ω scans	*h* = −6→9
Absorption correction: analytical (*CrysAlis PRO*; Agilent, 2010)	*k* = −10→10
*T*_min_ = 0.827, *T*_max_ = 0.951	*l* = −14→14
4547 measured reflections	

### Refinement

**Table:**

Refinement on *F*^2^	Secondary atom site location: difference Fourier map
Least-squares matrix: full	Hydrogen site location: inferred from neighbouring sites
*R*[*F*^2^ > 2σ(*F*^2^)] = 0.034	H-atom parameters constrained
*wR*(*F*^2^) = 0.096	*w* = 1/[σ^2^(*F*_o_^2^) + (0.0554*P*)^2^ + 0.167*P*] where *P* = (*F*_o_^2^ + 2*F*_c_^2^)/3
*S* = 1.05	(Δ/σ)_max_ = 0.001
2731 reflections	Δρ_max_ = 0.26 e Å^−^^3^
204 parameters	Δρ_min_ = −0.25 e Å^−^^3^
0 restraints	Extinction correction: *SHELXL97* (Sheldrick, 2008), Fc^*^=kFc[1+0.001xFc^2^λ^3^/sin(2θ)]^-1/4^
Primary atom site location: structure-invariant direct methods	Extinction coefficient: 0.0068 (13)

### Special details

**Table:**

Experimental. (CrysAlisPro; Agilent 2010)
Geometry. All e.s.d.'s (except the e.s.d. in the dihedral angle between two l.s. planes) are estimated using the full covariance matrix. The cell e.s.d.'s are taken into account individually in the estimation of e.s.d.'s in distances, angles and torsion angles; correlations between e.s.d.'s in cell parameters are only used when they are defined by crystal symmetry. An approximate (isotropic) treatment of cell e.s.d.'s is used for estimating e.s.d.'s involving l.s. planes.
Refinement. Refinement of *F*^2^ against ALL reflections. The weighted *R*-factor *wR* and goodness of fit *S* are based on *F*^2^, conventional *R*-factors *R* are based on *F*, with *F* set to zero for negative *F*^2^. The threshold expression of *F*^2^ > σ(*F*^2^) is used only for calculating *R*-factors(gt) *etc*. and is not relevant to the choice of reflections for refinement. *R*-factors based on *F*^2^ are statistically about twice as large as those based on *F*, and *R*- factors based on ALL data will be even larger.

### Fractional atomic coordinates and isotropic or equivalent isotropic displacement parameters (Å^2^)

**Table:**

	*x*	*y*	*z*	*U*_iso_*/*U*_eq_	
O20	0.15945 (10)	0.37481 (10)	0.32775 (6)	0.0189 (2)	
O17	0.09397 (11)	0.16999 (10)	0.13913 (7)	0.0215 (2)	
O2	1.52912 (10)	1.04374 (10)	0.31139 (7)	0.0209 (2)	
O14	0.37423 (11)	0.19633 (11)	0.00966 (7)	0.0228 (2)	
O4	1.43214 (11)	1.16372 (11)	0.45157 (7)	0.0249 (2)	
C19	0.31151 (15)	0.40188 (14)	0.26702 (9)	0.0164 (2)	
C22	0.49386 (15)	0.52658 (14)	0.29919 (9)	0.0164 (2)	
H22	0.5177	0.6002	0.3661	0.020*	
C16	0.27386 (15)	0.29495 (14)	0.16769 (9)	0.0174 (2)	
C6	1.17401 (15)	0.76841 (15)	0.27483 (9)	0.0185 (2)	
H6	1.2780	0.7473	0.2559	0.022*	
C7	0.99186 (15)	0.64548 (14)	0.24261 (9)	0.0183 (2)	
H7	0.9724	0.5398	0.2030	0.022*	
C11	0.64163 (15)	0.54313 (14)	0.23285 (9)	0.0166 (2)	
C10	1.05099 (15)	0.95224 (14)	0.36160 (9)	0.0191 (2)	
H10	1.0707	1.0566	0.4032	0.023*	
C5	1.20468 (15)	0.92276 (14)	0.33487 (9)	0.0168 (2)	
C8	0.83642 (15)	0.67484 (14)	0.26762 (9)	0.0164 (2)	
C18	0.00490 (16)	0.20396 (16)	0.04195 (11)	0.0254 (3)	
H18A	0.0834	0.2196	−0.0221	0.038*	
H18B	−0.0109	0.3094	0.0586	0.038*	
H18C	−0.1187	0.1061	0.0230	0.038*	
C9	0.86947 (15)	0.83046 (14)	0.32792 (10)	0.0190 (2)	
H9	0.7660	0.8529	0.3460	0.023*	
C3	1.39732 (15)	1.05633 (14)	0.37365 (9)	0.0180 (2)	
C13	0.42300 (16)	0.31014 (14)	0.10261 (9)	0.0183 (2)	
C15	0.51955 (16)	0.20895 (16)	−0.06177 (10)	0.0235 (3)	
H15A	0.4656	0.1228	−0.1263	0.035*	
H15B	0.6151	0.1876	−0.0190	0.035*	
H15C	0.5778	0.3252	−0.0890	0.035*	
C12	0.60543 (15)	0.43431 (14)	0.13456 (9)	0.0181 (2)	
H12	0.7057	0.4451	0.0894	0.022*	
C21	0.19948 (16)	0.44995 (16)	0.44155 (9)	0.0225 (3)	
H21A	0.2520	0.5762	0.4413	0.034*	
H21B	0.2908	0.4186	0.4804	0.034*	
H21C	0.0831	0.4065	0.4809	0.034*	
C1	1.72008 (15)	1.17042 (15)	0.34262 (11)	0.0229 (3)	
H1A	1.8043	1.1574	0.2888	0.034*	
H1B	1.7580	1.1529	0.4188	0.034*	
H1C	1.7275	1.2870	0.3415	0.034*	

### Atomic displacement parameters (Å^2^)

**Table:**

	*U*^11^	*U*^22^	*U*^33^	*U*^12^	*U*^13^	*U*^23^
O20	0.0124 (4)	0.0220 (4)	0.0179 (4)	0.0041 (3)	0.0012 (3)	−0.0038 (3)
O17	0.0139 (4)	0.0212 (4)	0.0202 (4)	−0.0003 (3)	−0.0014 (3)	−0.0019 (3)
O2	0.0114 (4)	0.0209 (4)	0.0239 (4)	0.0017 (3)	0.0009 (3)	−0.0050 (3)
O14	0.0173 (4)	0.0238 (4)	0.0196 (4)	0.0029 (3)	0.0015 (3)	−0.0086 (3)
O4	0.0183 (4)	0.0222 (4)	0.0269 (4)	0.0034 (3)	−0.0004 (3)	−0.0090 (3)
C19	0.0137 (5)	0.0174 (5)	0.0174 (5)	0.0061 (4)	0.0014 (4)	0.0017 (4)
C22	0.0148 (5)	0.0159 (5)	0.0158 (5)	0.0048 (4)	−0.0011 (4)	−0.0018 (4)
C16	0.0125 (5)	0.0158 (5)	0.0185 (5)	0.0016 (4)	−0.0017 (4)	−0.0010 (4)
C6	0.0139 (5)	0.0210 (5)	0.0191 (5)	0.0067 (4)	0.0015 (4)	−0.0018 (4)
C7	0.0160 (5)	0.0177 (5)	0.0180 (5)	0.0052 (4)	0.0000 (4)	−0.0045 (4)
C11	0.0141 (5)	0.0157 (5)	0.0179 (5)	0.0048 (4)	−0.0008 (4)	0.0007 (4)
C10	0.0173 (5)	0.0162 (5)	0.0211 (5)	0.0052 (4)	0.0023 (4)	−0.0022 (4)
C5	0.0141 (5)	0.0174 (5)	0.0150 (5)	0.0035 (4)	0.0004 (4)	0.0004 (4)
C8	0.0148 (5)	0.0167 (5)	0.0146 (5)	0.0040 (4)	0.0005 (4)	0.0005 (4)
C18	0.0174 (5)	0.0251 (6)	0.0294 (6)	0.0066 (5)	−0.0066 (5)	−0.0042 (5)
C9	0.0144 (5)	0.0183 (5)	0.0227 (6)	0.0058 (4)	0.0021 (4)	−0.0011 (4)
C3	0.0160 (5)	0.0175 (5)	0.0184 (5)	0.0056 (4)	0.0011 (4)	0.0004 (4)
C13	0.0185 (5)	0.0178 (5)	0.0158 (5)	0.0059 (4)	−0.0006 (4)	−0.0019 (4)
C15	0.0214 (6)	0.0264 (6)	0.0200 (6)	0.0084 (5)	0.0031 (4)	−0.0054 (5)
C12	0.0143 (5)	0.0193 (5)	0.0183 (5)	0.0055 (4)	0.0013 (4)	−0.0006 (4)
C21	0.0173 (5)	0.0282 (6)	0.0176 (6)	0.0063 (5)	0.0027 (4)	−0.0032 (4)
C1	0.0120 (5)	0.0201 (6)	0.0298 (6)	0.0012 (4)	0.0000 (4)	−0.0011 (5)

### Geometric parameters (Å, º)

**Table:**

O20—C19	1.3667 (13)	C10—H10	0.9500
O20—C21	1.4298 (13)	C10—C5	1.3916 (15)
O17—C16	1.3733 (13)	C10—C9	1.3851 (16)
O17—C18	1.4345 (14)	C5—C3	1.4891 (15)
O2—C3	1.3424 (13)	C8—C9	1.3997 (15)
O2—C1	1.4442 (13)	C18—H18A	0.9800
O14—C13	1.3618 (13)	C18—H18B	0.9800
O14—C15	1.4271 (13)	C18—H18C	0.9800
O4—C3	1.2076 (14)	C9—H9	0.9500
C19—C22	1.3937 (15)	C13—C12	1.3921 (15)
C19—C16	1.3969 (15)	C15—H15A	0.9800
C22—H22	0.9500	C15—H15B	0.9800
C22—C11	1.3972 (15)	C15—H15C	0.9800
C16—C13	1.4000 (16)	C12—H12	0.9500
C6—H6	0.9500	C21—H21A	0.9800
C6—C7	1.3881 (15)	C21—H21B	0.9800
C6—C5	1.3937 (15)	C21—H21C	0.9800
C7—H7	0.9500	C1—H1A	0.9800
C7—C8	1.3994 (15)	C1—H1B	0.9800
C11—C8	1.4857 (14)	C1—H1C	0.9800
C11—C12	1.3971 (15)		
			
C19—O20—C21	116.33 (8)	H18A—C18—H18B	109.5
C16—O17—C18	113.77 (9)	H18A—C18—H18C	109.5
C3—O2—C1	115.40 (8)	H18B—C18—H18C	109.5
C13—O14—C15	117.61 (9)	C10—C9—C8	120.93 (10)
O20—C19—C22	123.79 (10)	C10—C9—H9	119.5
O20—C19—C16	115.53 (9)	C8—C9—H9	119.5
C22—C19—C16	120.67 (10)	O2—C3—C5	111.90 (9)
C19—C22—H22	120.1	O4—C3—O2	123.63 (10)
C19—C22—C11	119.89 (10)	O4—C3—C5	124.47 (10)
C11—C22—H22	120.1	O14—C13—C16	115.08 (10)
O17—C16—C19	119.67 (10)	O14—C13—C12	124.58 (10)
O17—C16—C13	121.07 (10)	C12—C13—C16	120.34 (10)
C19—C16—C13	119.14 (10)	O14—C15—H15A	109.5
C7—C6—H6	119.9	O14—C15—H15B	109.5
C7—C6—C5	120.16 (10)	O14—C15—H15C	109.5
C5—C6—H6	119.9	H15A—C15—H15B	109.5
C6—C7—H7	119.5	H15A—C15—H15C	109.5
C6—C7—C8	121.03 (10)	H15B—C15—H15C	109.5
C8—C7—H7	119.5	C11—C12—H12	119.9
C22—C11—C8	119.97 (10)	C13—C12—C11	120.23 (10)
C12—C11—C22	119.69 (10)	C13—C12—H12	119.9
C12—C11—C8	120.34 (10)	O20—C21—H21A	109.5
C5—C10—H10	119.8	O20—C21—H21B	109.5
C9—C10—H10	119.8	O20—C21—H21C	109.5
C9—C10—C5	120.44 (10)	H21A—C21—H21B	109.5
C6—C5—C3	122.00 (10)	H21A—C21—H21C	109.5
C10—C5—C6	119.29 (10)	H21B—C21—H21C	109.5
C10—C5—C3	118.69 (10)	O2—C1—H1A	109.5
C7—C8—C11	120.91 (10)	O2—C1—H1B	109.5
C7—C8—C9	118.14 (10)	O2—C1—H1C	109.5
C9—C8—C11	120.95 (10)	H1A—C1—H1B	109.5
O17—C18—H18A	109.5	H1A—C1—H1C	109.5
O17—C18—H18B	109.5	H1B—C1—H1C	109.5
O17—C18—H18C	109.5		
			
O20—C19—C22—C11	178.85 (10)	C7—C6—C5—C10	−0.25 (17)
O20—C19—C16—O17	−1.73 (15)	C7—C6—C5—C3	178.45 (10)
O20—C19—C16—C13	−177.75 (10)	C7—C8—C9—C10	0.19 (17)
O17—C16—C13—O14	1.62 (16)	C11—C8—C9—C10	179.15 (10)
O17—C16—C13—C12	−178.14 (10)	C10—C5—C3—O2	−157.96 (10)
O14—C13—C12—C11	−178.77 (10)	C10—C5—C3—O4	21.96 (17)
C19—C22—C11—C8	−179.40 (9)	C5—C6—C7—C8	1.18 (17)
C19—C22—C11—C12	−0.02 (16)	C5—C10—C9—C8	0.72 (17)
C19—C16—C13—O14	177.58 (10)	C8—C11—C12—C13	179.52 (10)
C19—C16—C13—C12	−2.18 (17)	C18—O17—C16—C19	111.07 (11)
C22—C19—C16—O17	178.33 (9)	C18—O17—C16—C13	−72.99 (13)
C22—C19—C16—C13	2.31 (17)	C9—C10—C5—C6	−0.70 (17)
C22—C11—C8—C7	148.15 (11)	C9—C10—C5—C3	−179.43 (10)
C22—C11—C8—C9	−30.78 (15)	C15—O14—C13—C16	178.23 (10)
C22—C11—C12—C13	0.14 (16)	C15—O14—C13—C12	−2.02 (16)
C16—C19—C22—C11	−1.23 (16)	C12—C11—C8—C7	−31.23 (16)
C16—C13—C12—C11	0.97 (17)	C12—C11—C8—C9	149.84 (11)
C6—C7—C8—C11	179.90 (10)	C21—O20—C19—C22	−15.21 (15)
C6—C7—C8—C9	−1.14 (16)	C21—O20—C19—C16	164.86 (10)
C6—C5—C3—O2	23.34 (15)	C1—O2—C3—O4	−0.72 (16)
C6—C5—C3—O4	−156.74 (12)	C1—O2—C3—C5	179.20 (9)

### Hydrogen-bond geometry (Å, º)

**Table:**

*D*—H···*A*	*D*—H	H···*A*	*D*···*A*	*D*—H···*A*
C15—H15*A*···O2^i^	0.98	2.58	3.4933 (15)	156
C18—H18*C*···O14^ii^	0.98	2.50	3.4453 (16)	161

Symmetry codes: (i) −*x*+2, −*y*+1, −*z*; (ii) −*x*, −*y*, −*z*.
